# Concerns, attitudes, and intended practices of Caribbean healthcare workers concerning COVID-19 vaccination: A cross-sectional study

**DOI:** 10.1016/j.lana.2022.100193

**Published:** 2022-05

**Authors:** E. Benjamin Puertas, Martha Velandia-Gonzalez, Lauren Vulanovic, Lisa Bayley, Karen Broome, Claudia Ortiz, Nina Rise, Maite Vera Antelo, Dale A. Rhoda

**Affiliations:** a Pan American Health Organization, Office of the Subregional Program Coordination, Caribbean, Bridgetown, Barbados; b Pan American Health Organization, Family, Health Promotion and Life Course Department, Comprehensive Family Immunization Unit, Washington, DC, USA; c Biostat Global Consulting, Worthington, OH, USA

**Keywords:** Vaccine hesitancy, Healthcare workers, Caribbean, COVID-19, Survey, Vaccine acceptance

## Abstract

**Background:**

The Caribbean has a long history of being a global leader in immunization, and one factor contributing to this success has been the commitment of healthcare workers in promoting the benefits of vaccines. Healthcare workers play a critical role in building trust between the public and the immunization program and are generally cited as the most trusted source of information on vaccination. Healthcare workers themselves, therefore, must be confident in vaccination as a public health good and able to transmit this confidence to those who trust them. However, just as with the general public, healthcare workers develop confidence at different rates and may be susceptible to misinformation about vaccines.

**Methods:**

During April and May 2021, the Pan American Health Organization (PAHO) conducted a mixed-methods survey to assess vaccination attitudes, opinions, and reasoning of 1197 healthcare workers across 14 Caribbean countries.

**Findings:**

Seventy-seven percent of respondents expressed clear intention to be vaccinated for COVID-19 as soon as possible. Intention to be vaccinated as soon as possible was expressed by lower proportions of nurses (66%) and allied health professionals (62%) than physicians (85%) and by younger respondents than older ones (64% vs. 85%, respectively; *p* < 0.001 for all these comparisons). Across 32 questions about attitudes and opinions, vaccine hesitancy was consistently expressed by higher proportions of nurses and allied health professionals than physicians and by younger respondents than older ones.

**Interpretation:**

Insights from the survey are helping PAHO address healthcare worker concerns with informative messages and supporting countries in policy development to increase vaccine confidence and coverage among Caribbean healthcare workers.

**Funding:**

This work has been sponsored by the World Health Organization/Pan American Health Organization, the Government of Germany and The Gavi Alliance.

**Research in context**

**Evidence before this study**

In early 2021 no other studies had examined the attitudes of Caribbean healthcare workers to COVID-19 vaccination, but long experience with attitudes toward influenza vaccine and healthcare worker surveys in other parts of the world sensitized PAHO leaders to the likelihood of a variety of attitudes and opinions. Feedback through informal professional channels supported the notion that some healthcare workers were likely to be hesitant toward forthcoming vaccines for COVID-19 vaccine.

**Added value of this study**

This study included respondents from 14 countries in proportions intended to be in line with the number of healthcare workers there and used both Likert-type scale response questions and open-text opinion questions to probe attitudes toward vaccines in general and forthcoming COVID-19 vaccines in particular. It found that 77% of Caribbean healthcare workers intended to be vaccinated as soon as possible, but 23% did not. The survey identified nurses (34%), allied health professionals (38%), and younger workers (85% of age quartile 51–87) as more likely to be hesitant than their counterparts. These insights have helped PAHO craft a regional policy statement and targeted communication to address the concerns of these visible, vocal, influential members of Caribbean society.

**Implications of all the available evidence**

While most Caribbean healthcare workers are confident enough to be vaccinated and to recommend vaccination, it is worthwhile to address the specific concerns of those who are hesitant in language that meets their needs and using messengers and channels they are likely to respect.

Alt-text: Unlabelled box

## Introduction

Healthcare workers (HCWs) are at the forefront of the COVID-19 pandemic. They provide care and comfort while taking on increased risk of infection, hospitalization, and death themselves. They are indeed heroes.

Vaccines present an important measure for gaining control of the COVID-19 pandemic and research has been occurring at an accelerated rate to provide safe, effective vaccines.^1^^,^^2^ In September 2020, the World Health Organization (WHO) Strategic Advisory Group of Experts on Immunization (SAGE) released a Values Framework for the Allocation and Prioritization of COVID-19 Vaccination suggesting that countries should prioritize HCWs, older adults, and adults with chronic diseases to receive the first doses of an approved COVID-19 vaccine.^3^ Healthcare workers are among the public's most trusted sources of information on vaccines and vaccination.^4^ But not all HCWs eagerly accept or promote COVID-19 vaccines. Studies from around the world have found nurses to be more hesitant towards COVID-19 vaccination than other HCWs.^5^, ^6^, ^7^ Women have been identified by several studies to be more vaccine hesitant than men.^6^^,^^8^, ^9^, ^10^, ^11^, ^12^, ^13^, ^14^ Studies from Latin America and the Caribbean have found rural-dwelling, lower education, and financial insecurity to be associated with vaccine hesitancy among both HCWs and the public.^12^^,^^15^ These findings agree with others found elsewhere.^11^^,^^13^^,^^16^

The Caribbean has a long history of being a global leader in immunization.^17^ A recent study conducted among British HCWs, finds those of Caribbean descent to be most COVID-19 vaccine hesitant (38.1% - 51-0%),^14^ but the rate of hesitance among Caribbean HCWs in the Caribbean has not been evaluated. This paper reports on a 2021 Internet-based survey conducted among 1197 healthcare workers from 14 Caribbean countries. The objectives were to gather and use quality data on behavioral and social drivers of vaccination and COVID-19 vaccines among HCWs in the Caribbean, and to inform implementation strategies and communication approaches on COVID-19 vaccines (and vaccines in general) in the Caribbean, with the final aim to contribute to increasing vaccination acceptance and improving vaccine confidence among healthcare workers.

COVID-19 vaccine acceptance among HCWs might be improved with targeted communication campaigns. Technology and social media are being used on an unprecedented scale to keep people safe, informed, productive, and connected. But the newly developed COVID-19 vaccines have also been significant targets of mis- and disinformation, leading to public mistrust and concern. Social media has been demonstrated to be a powerful channel for propagating anti-vaccine information and diminish uptake of vaccines.^18^, ^19^, ^20^ But when social media is used for good – to spread reliable vaccine information from trusted healthcare workers and public health authorities – it can foster public trust in vaccination.^18^

Targeting HCWs with helpful communication is important to increase vaccine uptake.^21^ Studies of HCWs in Europe and Canada found concerns about vaccine safety to be a key influencer of vaccine hesitancy.^22^^,^^23^ In France, vaccine information specifically targeting HCW have shown to increase vaccine uptake among hospital staff^24^ and HCWs’ trust in the institutions delivering information on vaccines and vaccination is essential for vaccine acceptance.^22^ A study among HCWs in Mexico found information and being well-informed to be keys to vaccine uptake.^25^

## Methods

### Survey instrument development

The questionnaire combined items from a WHO & UNICEF guidance document, and a questionnaire developed at the University of California at Los Angeles.^26^^,^^27^ It was adapted for use in the Caribbean, reviewed by the Caribbean Technical Advisory Group for Immunization, and piloted in the Caribbean to ensure questions and response options were understood as intended.^28^ Questions on the influenza vaccine were added to facilitate comparison between attitudes towards COVID-19 vaccine and another vaccine given to adults in the Caribbean. The survey instrument appears in Annex A in the electronic supplement.

Questions were grouped into several categories:

1. Personal and occupational demographics
2. Attitudes toward vaccines in general
  a. Attitudes to vaccines in general (7 Likert questions)
  b. Vaccine readiness (3 Likert questions)
3. Attitudes towards COVID-19 vaccines
  a. Overall attitudes (3 Likert questions)
  b. Vaccination if a COVID-19 vaccine becomes publicly available: (4 Likert questions)
  c. Reasons for delaying or refusing a COVID-19 vaccine: (5 Likert and one open-ended)
4. Factors contributing to opinions of COVID-19 vaccines (8 Likert questions and one open-ended)
5. Attitudes towards influenza vaccine (2 Likert questions and two open-ended)

The question “If a COVID-19 vaccine becomes available, I intend to get it as soon as possible” was selected as a proxy of COVID-19 vaccine acceptance. Those who disagreed or strongly disagreed with this statement were considered to be COVID-19 vaccine hesitant.

### Ethics committee and confidentiality

The study protocol was approved by the Pan American Health Organization (PAHO) Ethics Review Committee. Each respondent gave consent at the start of the questionnaire; each was informed they were free to take part in the research study or not with no negative consequences either way and there were no expected appropriate answers to the questions.

### Sample size

The target sample size for the study was calculated using the total number of HCWs in the categories reported to the WHO National Health Workforce Accounts Portal (NHWA): nurses, physicians, midwives, dentists, and pharmacists. Fourteen countries of the Caribbean reported a total of 38,671 HCWs; this was taken to be the size of the population of eligible respondents. To calculate the sample, a complex multilevel sample design was assumed. To be conservative, the inferential goal was to estimate Caribbean HCW vaccine hesitancy using a two-sided Wald-type margin of error no wider than ±5% if 50% of HCWs were hesitant and if complex sampling carried a design effect up to 2. The resulting sample size (*n* = 761) was allocated proportionally across countries as shown in Table 1. Sample size was calculated using OpenEpi v3.01.^29^ Although it was hoped that the call for participation would yield healthcare workers in the sample that followed roughly the same proportions as the population, no attempt was made to stratify the sample within countries and no limits were placed on the number of respondents from any eligible HCW category.

**Table 1:** Target sample size by country and number of respondents by country and job category.

	Observed Number of Respondents						Target Sample Size
	Physicians	Nurses	Public Health	Allied Pros	Other	Total	
Antigua and Barbuda	17	7	2	3	0	29	19
Bahamas	8	22	13	13	23	79	55
Barbados	43	13	8	13	5	82	41
Belize	9	21	6	6	5	47	33
Dominica	1	4	3	4	2	14	11
Grenada	7	25	5	4	2	43	19
Guyana	3	3	1	5	1	13	46
Haiti	59	18	16	7	2	102	102
Jamaica	151	18	16	27	3	215	87
St Kitts and Nevis	3	62	11	6	4	86	16
St Lucia	1	9	6	2	1	19	7
St Vincent and the Grenadines	1	16	2	3	2	24	18
Suriname	30	19	7	2	4	62	44
Trinidad and Tobago	188	93	20	63	18	382	263
Total	521	330	116	158	72	1,197	761

### Survey implementation

Data were collected anonymously using an Internet survey in English and in French via Qualtrics®^30^ which recorded the respondents’ start and end date and time, and used browser cookies, IP address tracking, and geocoordinates to prevent multiple submissions by the same respondent. Each respondent was presented with all the questions, but not required to respond to any opinion questions.

There was no review or confirmation step at the end of the survey. All Likert questions used four response options: Strongly agree; Agree; Disagree; Strongly disagree.

In Trinidad and Tobago, there were complications due to poor Internet access, so a paper form of the questionnaire was circulated. Paper forms were collected for 86 respondents and their responses were anonymously uploaded using Qualtrics at the PAHO country office (with stable Internet access).

PAHO and the national ministries of health advertised the survey through professional associations of the participating countries, through health clinics, professional associations, and societies as well as the Regional Nursing Body, the Caribbean Community (CARICOM), and academic institutions, specifically the University of West Indies (UWI), to be distributed to their list of graduate health care practitioners. Toward the end of the data collection period, some countries had not fulfilled their proportional share of the sample; officials there were asked to promote the survey again among networks of HCWs.

There were no payments or incentives to complete the survey. Data collection occurred between 15 March and 30 April 2021.

### Statistical analysis

Data were managed and analyzed using Microsoft Excel and Stata Release 17.^31^ Responses were summarized using simple unweighted proportions as if the data were from a simple random sample of Caribbean HCWs. Chi-square statistics and multivariable logistic regression were used to assess differences for every opinion question. For binary analyses, respondents who said ‘Strongly agree’ or ‘Agree’ were coded with an outcome of 1 and those who said ‘Disagree’ or ‘Strongly disagree’ were coded with 0. Explanatory factors for regression included three categorical variables: job category (five levels, with physicians as the reference group), sex (with males as the reference), and age quartile (with the youngest quartile as the reference group).

The question that was selected as a proxy for COVID-19 hesitancy (Q19) is one of several that might have been selected. To explore the relationships between that question and others, Pearson correlation coefficients were calculated between responses to Q19 and responses to every other Likert-response question.

It is well known that analyzing data from a complex sample design (or a complex convenience sample) as if it came from a simple random sample yields misleadingly small *p*-values and increased risk of Type I error.^32^^,^^33^ Rather than emphasize individual p-values, the study team looked for patterns of numerous concordant, apparently significant, adjusted odds ratios and interpreted those patterns as useful for identifying target demographics for communication strategies. Annex C in the electronic supplement summarizes responses and shows chi-square and logistic regression results for every opinion question in the questionnaire.

### Summarizing open-ended responses

Although most questions yielded quantitative responses, four open-ended questions provided opportunities for HCWs to describe reservations about vaccination.

Q28: Other reasons for delaying or refusing COVID-19 vaccine
Q37: Other factors that contributed to my opinion on a COVID-19 vaccine
Q39: If you disagree with taking the flu vaccine, why?
Q41: If you disagree with recommending the flu vaccine to friends and family, why?

Text responses to those questions were assessed using the WHO behavioral and social drivers (BeSD) of COVID-19 vaccination model, which was adapted by the Caribbean survey team, with guidance from WHO headquarters staff.^26^ French open-ended responses were translated automatically using Google Sheets and the Google Translate function. Both the French response and English translation were furnished to three pairs of investigators. The pairs categorized each response as reflecting one of four domains of the BeSD model: thinking and feeling, individual motivation, social processes, and practical issues, and further categorized which constructs of the domain were relevant. All pairs collated their work and conferred to resolve discordant categorizations. Some free text responses contained ideas that reflected two separate constructs, sometimes within two different domains. Those were coded as belonging to both constructs and domains. These responses contained important contextual information that complemented the quantitative results.

### Role of the funding source

This work has been sponsored by the World Health Organization/Pan American Health Organization, the Government of Germany and The Gavi Alliance. The funders did not have any role in design of the survey, interpretation of results, decision to publish, or drafting of manuscript.

## Results

**Figure 1:**
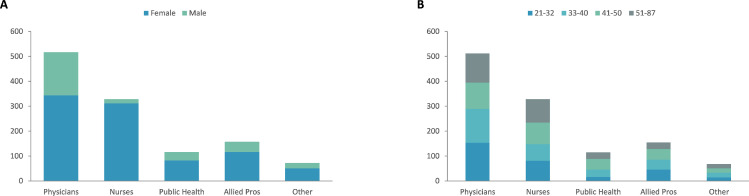
Respondents by age, sex, and job category. *Counts for Figure 1 appear in Annex B of the electronic supplement.

In total 1,197 HCWs completed the survey; all countries and territories except Guyana managed to fill (or more than fill) their suggested sample size. Table 1 summarizes respondents by country and job category. Figure 1 characterizes the sample showing gender and age by job category. The number of opinion questions answered by respondents varied from as few as 1 to as many as 32 (all); the mean was 26 and median was 28. The electronic supplement includes a post-hoc factor analysis to explore dimensionality of the questionnaire. Missing responses prevented calculating factor scores, but hesitancy came through as a clear theme in the first factor identified.

Table 2 summarizes the portion of respondents in various categories who said they agree or strongly agree with the survey's opinion questions. Concerning attitudes to vaccines, respondents displayed widespread agreement with 90% or more agreeing that vaccines are safe and efficient, and a good way to protect oneself from disease. They agreed that vaccine information provided by public health authorities and healthcare providers is reliable and trustworthy. All the sub-groups but one reported at least 90% agreement that they do what their health care provider recommends about vaccines. Nurses and allied health professionals showed lower agreement with that latter statement (91% and 87%, respectively) than physicians (94%); those differences were statistically significant after adjusting for age and gender (*p* = 0 × 044 and 0·033, respectively). While most of both genders (90–99%) agreed with the seven pro-vaccine attitude questions (Q6-Q12), females agreed even more than males. The gender differences were significant for five of those questions, after having controlled for differences in HCW job category and age quartiles. Table 2 also reports how the four-option Likert responses to each question were correlated with responses to the hesitancy proxy question, Q19. Intention to obtain the vaccine as soon as possible was strongly correlated (coefficient > 0.5) with similar questions like vaccine information being trustworthy, doing what the healthcare provider recommends about vaccines, recommending a COVID-19 vaccine to family and friends, and others.

**Table 2:** Summary of responses by HCW categories, age, and sex.

Questions about new vaccines, COVID-19 vaccines, and the factors that shape those opinions yielded many statistically significant differences between sub-groups of respondents. Note, in particular, the consistent pattern of nurses being more hesitant than physicians in Table 2 (22 of 32 rows), allied health professionals being more hesitant than physicians (19 of 32 rows) and the youngest age quartile being more hesitant than the oldest (15 of 32 rows).

### Vaccine hesitancy

**Figure 2:**
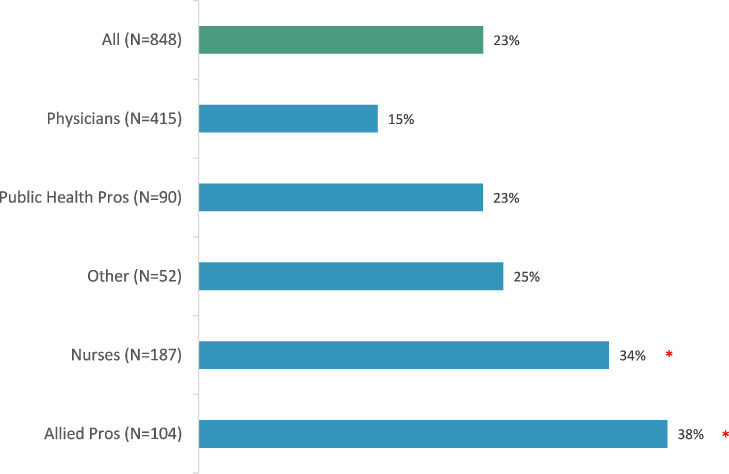
COVID-19 vaccine hesitancy by HCW category**.** % who disagree with the statement: "If a new COVID-19 vaccine becomes available, I intend to get it as soon as possible." *The portion of respondents disagreeing in this category differs from the portion of physicians by an amount that is statistically significant, after adjusting for worker sex and age.

When asked if they would take the vaccine as soon as possible, of 848 participants who answered the question, 195 (23%) respondents displayed some degree of hesitancy. Across HCW categories, 15% of physicians disagreed with getting a COVID-19 vaccine as soon as possible compared with 34% of nurses (*p* < 0·001), 23% of public health professionals (*p* = 0·014), 38% of allied professionals (*p* < 0·001), and 25% of other professionals (*p* = 0·089) (Figure 2 and **Annex C** of the electronic supplement).

**Figure 3:**
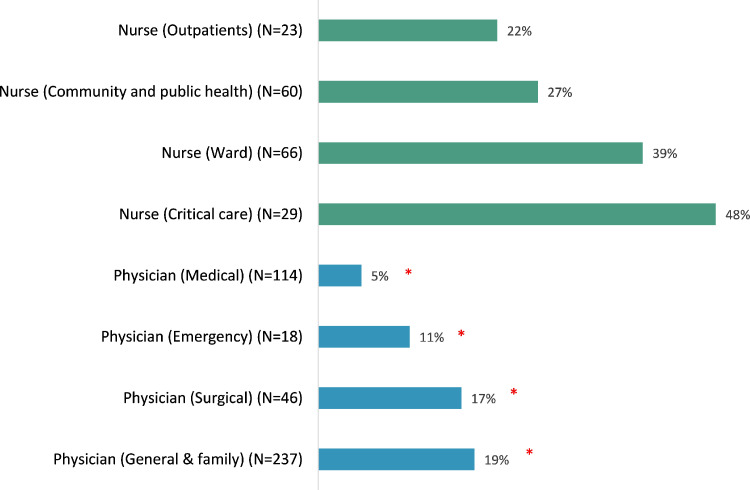
COVID-19 vaccine hesitancy by HCW subcategory (specialty). % who disagree with the statement: "If a new COVID-19 vaccine becomes available, I intend to get it as soon as possible." *Chi-square indicates statistically significant differences in hesitancy among categories of physicians, but not among categories of nurses.

Differences in hesitancy between sub-categories of nurses were not significant (chi-square *p* = 0·092). However, there were significant differences between physicians' specialties, with medical and surgical clinicians and emergency physicians being less hesitant compared to general practitioners and family physicians (chi-square *p* = 0·007) (Figure 3).

**Figure 4:**
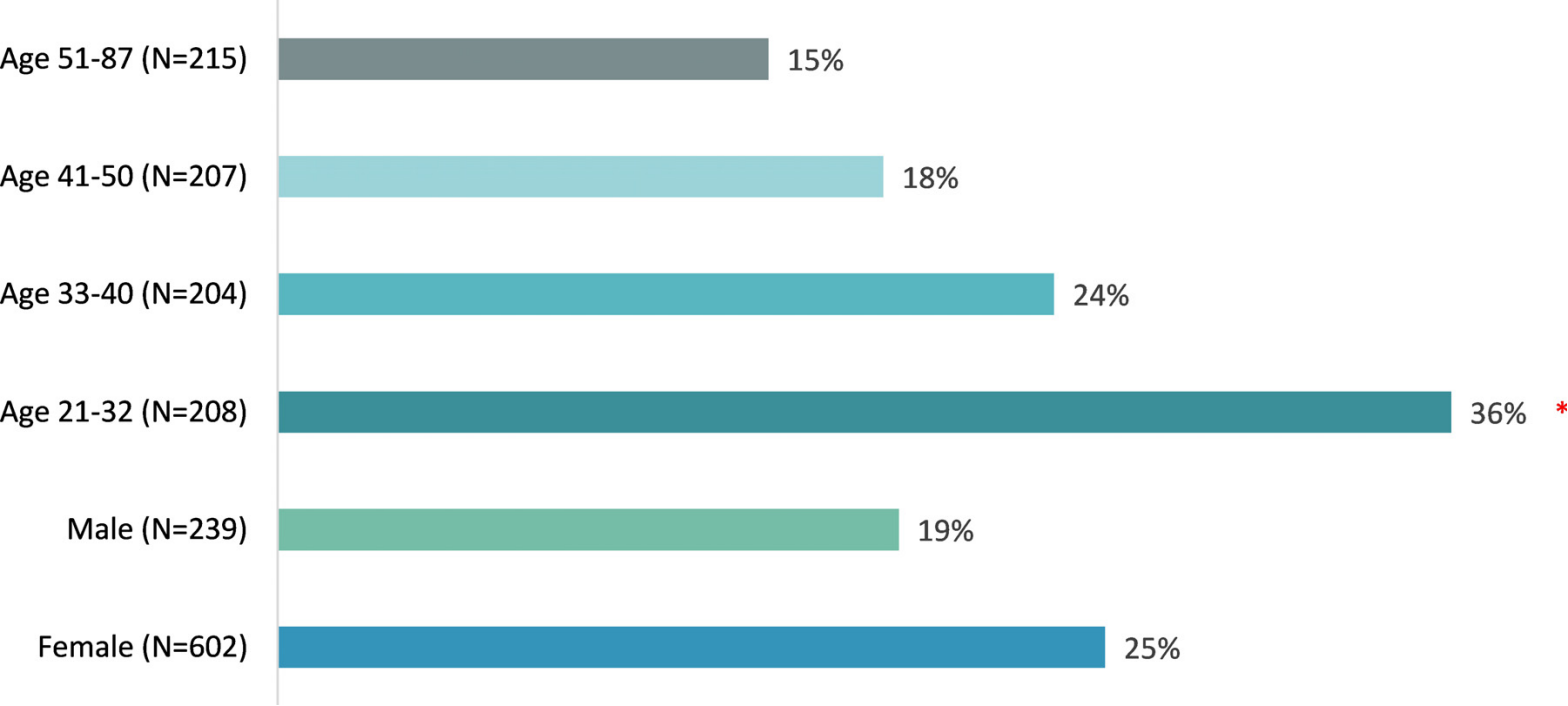
Vaccine hesitancy by HCW age group. % who disagree with the statement: "If a new COVID-19 vaccine becomes available, I intend to get it as soon as possible." *Logistic regression indicates that respondents in the youngest quartile were significantly more hesitant than those in any of the older quartiles, adjusting for job category and gender.

The difference between sexes was not significant, with 19% of males and 25% of females indicating hesitance (*p* = 0·731). When comparing across age quartiles (AQ), vaccine hesitancy was most prevalent among younger HCW, where only 64% of AQ 21-32, compared with 76% of AQ 33-40 (*p* = 0·007), 82% of AQ 41-50 (*p* < 0·001), and 85% of AQ 51-87 (*p* < 0·001) intended to get a COVID-19 vaccine as soon as possible (Figure 4).

A third of physicians (36%) wanted to wait to see how the COVID-19 vaccine affects others compared with 60% of nurses (*p* < 0·001), 59% of allied professionals (*p* < 0·001), and 58% of ‘others’ (*p* < 0·001). So did 61% of the youngest respondents, AQ 21–32, compared with 49% of AQ 33–40 (*p* = 0·011), 42% of AQ 41–50 (*p* < 0·001), and only 35% of AQ 51–87 (*p* < 0·001). Similarly, 29% of physicians compared with 52% of nurses (*p* < 0·001), 51% of allied professionals (*p* < 0·001), and 47% of others (*p* = 0·005) agreed that while they did not intend to get a COVID-19 vaccine soon, they might in the future. So did 47% of the youngest respondents, AQ 21–32, compared with 31% of the oldest respondents, AQ 51–87 (*p* < 0·001). Only 4% of all participants stated an intention to refuse a COVID-19 vaccine altogether, comparing across gender 8% of male respondents compared with 3% of female respondents agreed that they did not intend to ever get a COVID-19 vaccine (*p* < 0·001) (Table 2).

A third of participating healthcare workers did not know enough about the vaccines to make a decision whether to be vaccinated, mostly critical care nurses and allied health professionals (*p* < 0.001) in the younger age groups. Almost half of respondents (47%) agreed or strongly agreed that the development of the vaccine may have been rushed or that the vaccine may not have been thoroughly tested, with more nurses agreeing with that statement (60%, *p* < 0.001).

### Reasons to avoid or delay COVID-19 vaccination and opinion shapers

Responses were more hesitant for nurses than physicians for all five questions about reasons to delay COVID-19 vaccination and for five of the eight questions about opinion-shaping factors. Allied health professionals also showed elevated hesitancy as did the youngest compared with oldest. Nurses and young workers also relied more on opinion of family and friends and on social media than physicians and older respondents.

30% of respondents agreed that they do not yet know enough about the vaccine to decide; however, this was true for only 20% of physicians compared with 45% of nurses (*p* < 0.001), 45% of allied professionals (*p* < 0.001) and 35% of ‘other’ HCWs (*p* = 0.008). For 48% of respondents, the country of manufacture of a COVID-19 vaccine shaped their opinion on the vaccine. Among physicians, 46% agreed with this statement, compared with 57% of nurses (*p* = 0.004). 30% of respondents reported that information they have seen on social media shaped their opinion of a COVID-19 vaccine. This was true for only 21% of physicians, compared with 43% of nurses (*p* < 0.001), 35% of allied professionals (*p* = 0.002), and 39% of ‘other’ HCWs (*p* = 0.006). 38% of AQ 21–32 agreed that social media shaped their opinion on COVID-19 vaccine, compared with 30% of AQ 41–51 (*p* = 0.030) and 23% of AQ 51–87 (*p* < 0.001).

### Qualitative responses

**Figure 5:**
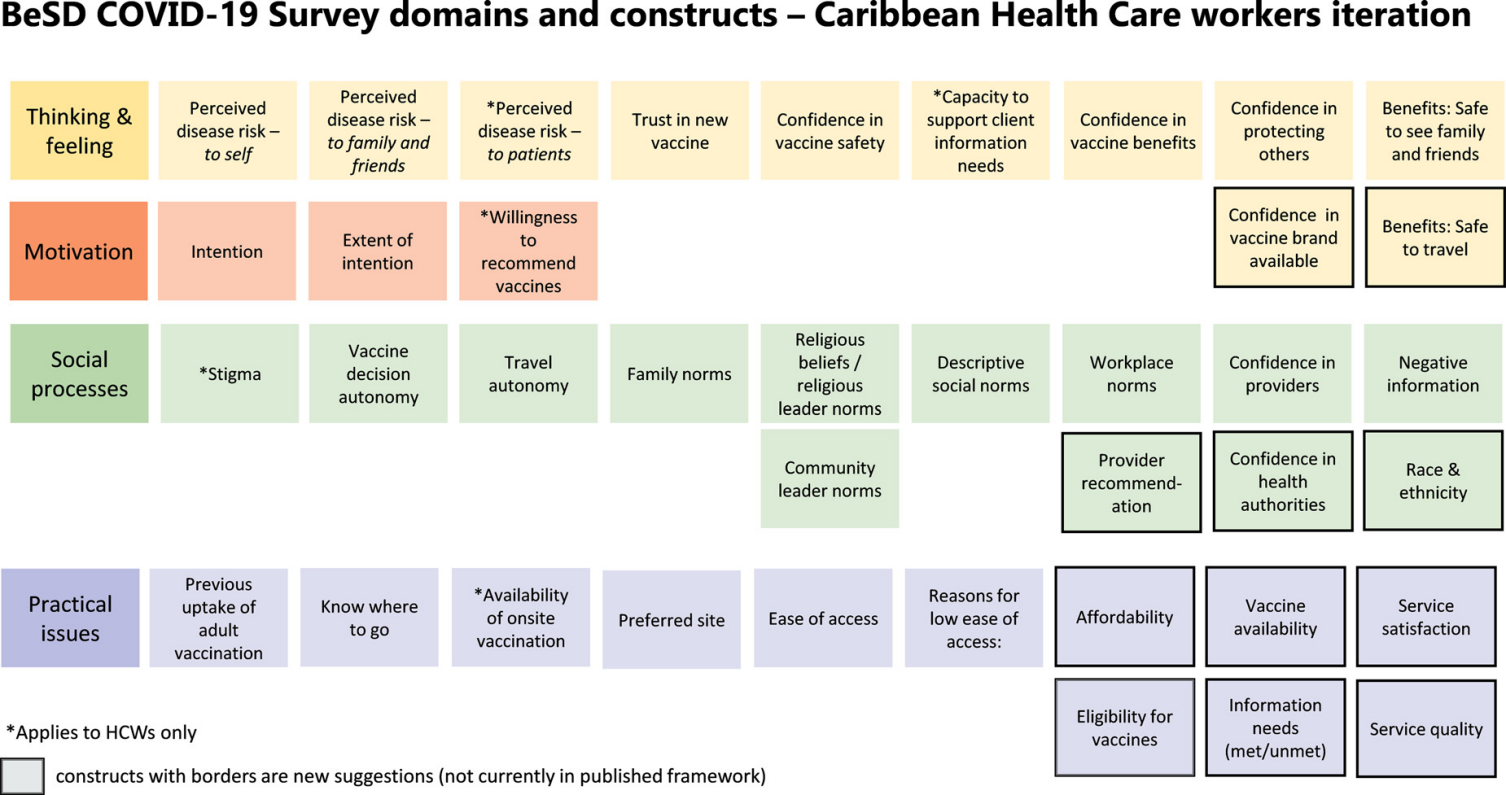
WHO behavior and social determinants domains and constructs for COVID-19 vaccines – Caribbean HCWs survey iteration**.**

**Figure 6:**
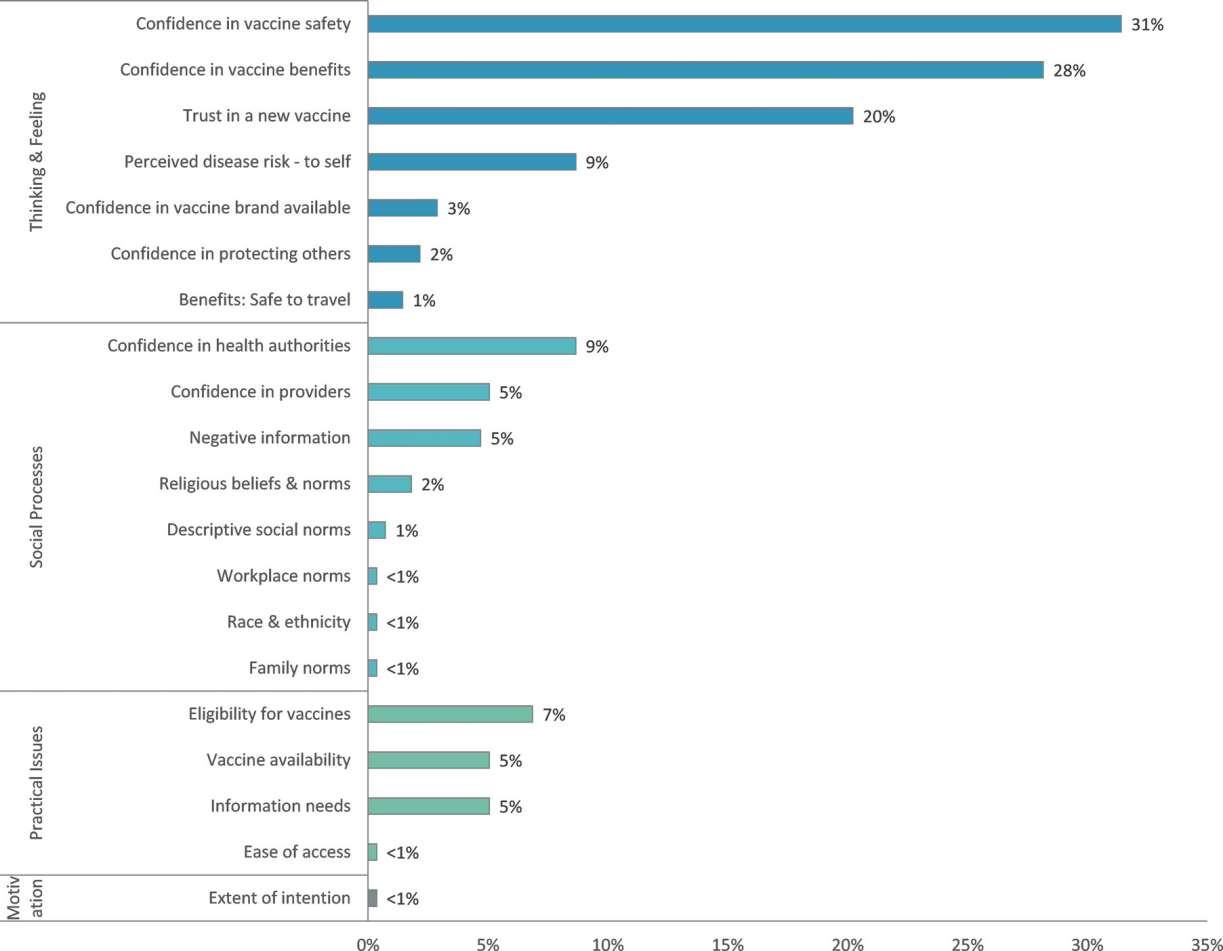
Qualitative response domains and constructs classified using the WHO Behavioral and Social Drivers (BeSD) model – open-ended questions about COVID Vaccines. Open text answers from *N* = 277 respondents of Q.28 and/or 37 were categorized for this figure.

Figure 5 shows the domains and constructs in the WHO BeSD tool and 11 that were added as part of this survey exercise. As seen in Figure 6, the addition of the “confidence in health authorities” construct figured prominently in the “social processes” BeSD domain, with “confidence in vaccine brand available” and “safe to travel” both being added to the “thinking and feeling” domain. For the “practical issues” BeSD domain, constructs related to vaccine affordability, availability, and eligibility; service satisfaction and quality; and information needs were added. The survey team identified free-text responses pertaining to COVID-19 that represent 20 of the 39 constructs listed in Figure 5.

### Attitude towards COVID-19 vaccines

Regarding the two questions related to HCWs´ opinions on COVID-19 vaccines (Q28 “Other reasons for delaying or refusing a COVID-19 vaccine” and Q37 “Other factors in my COVID-19 vaccine opinion”), the respondents´ answers overwhelmingly aligned with the BeSD thinking and feeling domain. The primary construct identified as part of the qualitative analysis was related to doubts regarding vaccine safety (31%). Many respondents pointed to their concerns regarding potential long-term side effects caused by the vaccines as a reason for influencing their opinion and for refusing or delaying the COVID-19 vaccine. Similarly, an important number of answers within the thinking and feeling domain fell under the construct related to confidence in vaccine benefits (28%). These answers pointed to sentiments of uncertainty on the length of the immunity provided by the vaccine, as well as the protection (or lack thereof) against variants of concern. Another important and significant construct that the respondents reported was related to trust (or lack thereof) in the COVID-19 vaccines (20%) (Figure 6). Some respondents argued that a low prevalence of the disease in their country at the time the survey was available rendered the COVID-19 vaccines unnecessary. Others suggested that if a different brand of the COVID-19 vaccines were made available, their intent of getting vaccinated would change toward vaccine acceptance.

Among the responses classified under the social processes’ domain, the most influential number of answers involved HCWs confidence (or lack thereof) in their health authorities. Respondents voiced concerns on issues such as authorities´ handling of the pandemic and the messaging communicated to the public. This issue is exacerbated when combined with vaccine safety concerns. Issues related to global equity appeared in some responses, with participants indicating skepticism that their countries would already be receiving quality vaccines. Some HCWs reported negative information as influencing their opinion but on the other hand, other HCWs pointed to a lack of information as influencing their opinion on the COVID-19 vaccines.

Some respondents listed pregnancy as a reason for not wanting to get vaccinated; since COVID-19 vaccination was not offered to pregnant individuals in all countries at the time of the survey, it is unclear whether respondents were referring to practical issues (i.e., they would like to get vaccinated but were unable to because vaccination was not being offered to pregnant individuals at that time) or if they were referring to an increased risk perception toward taking the vaccine.

Finally, although it was not an answer that appeared often, some respondents raised racial concerns, indicating that the vaccines had not been properly tested in all races and ethnicities, and therefore might not be safe for the Caribbean population. One participant noted: “Based on past ethical issues black people do have some trust issues which must be addressed to give more confidence in vaccines.”

## Discussion

Vaccination is one of public health´s most effective tools for protecting populations from many dangerous diseases, including now against COVID-19; however, some HCWs are not fully convinced of the effectiveness and safety of these new vaccines, which can result in a delay or refusal to get vaccinated when offered. Healthcare workers are the first priority population for vaccination against COVID-19, as established by SAGE in the roadmap for prioritizing uses of COVID-19 vaccines in the context of limited supply, and they are the most trusted source of vaccine and vaccination-related information to the general population.^3^^,^^34^ Eighty-eight percent of respondents said they would recommend a COVID-19 vaccine to friends and family. The concerns, attitudes and intended practices of physicians, nurses, and other healthcare workers influence the social and behavioral drivers of vaccination among the general public. Formerly hesitant workers who research the issues and decide to be vaccinated may be especially relatable and persuasive to patients or family members who have lingering doubts.^35^

In this study assessing the intention of healthcare workers to get the COVID-19 vaccine as soon as possible, 77% of the participants would receive the vaccine and 23% could be qualified as vaccine hesitant. However, despite 23% of respondents indicating they would not get vaccinated against COVID-19 as soon as they had the opportunity, only 4% of respondents reported that they never intend to get vaccinated. Compared to other healthcare workers in the Americas, Caribbean healthcare workers are less hesitant than their colleagues in French Guiana, where 66.4% of healthcare workers were willing to be, or had already been, vaccinated against COVID-19.^36^ Nurses were classified as hesitant at a rate twice more than physicians, and younger age quartiles reported more hesitancy to COVID-19 vaccination than older age groups. Our findings are consistent with similar studies carried out elsewhere.^6^^,^^13^^,^^23^^,^^27^

We also found important differences among specialties within professional categories, especially physicians and nurses. Clinicians and emergency physicians were more prone to want to get the vaccine as soon as possible, compared to general practitioners and family doctors. As Verger et al. noted, HCWs are not a homogeneous group, and most are not immunization experts, which is why building trust in this population requires providing credible information from trustworthy sources.^22^

Regarding gender, other than some small but statistically significant difference concerning attitudes, the study did not find consistent differences in responses between male and women, an association that has been found in various papers.^6^^,^^27^^,^^37^ We identified higher willingness for uptake of a COVID-19 vaccine in the oldest age group, which, at the time of the survey, was the most vulnerable group in terms of suffering severe outcomes from COVID-19.^27^

The BeSD model can provide insight about vaccine uptake categorized into four related domains: how people think and feel about vaccines; social processes that can encourage or discourage vaccine uptake; individual´s motivations to get vaccination – and in the case of HCWs, to recommend vaccination - and practical issues that can facilitate or hinder vaccination. Qualitative analysis of open-ended questions using the BeSD model confirmed and complemented the quantitative findings, with respondents expressing concerns related to perceived risks –including in the long-term– with taking COVID-19 vaccines, doubts regarding the vaccines´ ability to effectively protect against COVID, and a lack of information from trusted sources or lack of trust in authorities as the study of Verger et al. reported.^22^ Concerns for vaccination safety were common findings in other studies.^22^^,^^37^ There were some contrasting responses regarding vaccine safety. Gaps in perceived risk of COVID-19 vaccination versus risk of COVID-19 disease were widened by some respondents who answered that they did not see COVID-19 as a problem in their countries. The most repeated constructs mentioned by respondents (33%) were related to confidence in vaccine safety, benefits, and trust in new vaccines. In addition to the constructs already established in the WHO BeSD document,^26^ eleven new constructs were identified among Caribbean HCW responses, including one expressing that respondents would be more inclined to accept vaccination if their preferred vaccine brand was available. Figure 5 highlights all eleven new constructs. The most influential construct under the social processes' domain was the lack of HCW confidence in their health authorities (10%), with participants including statements in the open-ended questions on mandating vaccines, dishonesty, and denial or hiding of adverse events.

### Using the results to engage Caribbean healthcare workers

Efforts need to be made to increase risk perception of COVID-19 disease versus the approved vaccines so HCWs will feel more confident not only getting vaccinated themselves, but also in recommending that their patients, family, and friends get vaccinated. Strategic messaging should emphasize the importance of taking the first vaccine that´s available and not delaying vaccination in hopes of receiving a vaccine of personal preference. Trusted spokespeople should be used to empathetically communicate important messages about vaccine safety and efficacy and the importance of getting vaccinated with the first vaccine that is offered. This is especially important in situations where health authorities and government figures are not trusted sources of information for all audiences. References to religious objections for not being vaccinated, as indicated in some qualitative responses, highlight the need to work with religious leaders among these trusted spokespeople.

The WHO BeSD framework outlines possible interventions to be implemented at country level to improve vaccine acceptance among HCWs.^26^ In October 2021, WHO assembled a list of evidence-based interventions to increase vaccine uptake based on which BeSD domains are identified as important in the results.^38^ Additionally, the survey results have informed a regional policy brief proposal whose components address vaccine hesitancy; its recommendation and policy actions are meant to be adopted and adapted at the country level.^39^

Given answers to open-ended questions that allergies, previous infection with COVID-19 or underlying medical conditions were reasons for not immediately being vaccinated against COVID-19, messaging should also seek to clarify that allergies are not a contraindication for vaccination, and that many co-morbidities in fact increase the risk of complications from COVID-19 disease, meaning populations with those conditions will benefit greatly from the protection offered by COVID-19 vaccines. Additionally, the importance of getting vaccinated against COVID-19 following prior infection should be clearly communicated.

Likewise, in response to participants´ responses about not having enough information or not enough research having been carried out to make sound decisions about COVID-19 vaccination, results of studies should be clearly and transparently communicated and explained to HCWs, so they are continuously informed about new findings on vaccine effectiveness and safety.

Considering the statistically significant hesitancy among respondents in the youngest age group, a variety of channels should be employed to reach this audience with key messages in favor of vaccination. For example, authorities should explore social media platforms like Instagram and Tiktok in addition to traditional communications channels.

Reasons for vaccine hesitancy listed in the qualitative answers that can be classified as misinformation, as well as the indication that social media is a source of information for HCW about COVID-19 vaccines, show that HCW would benefit from targeted training on identifying misinformation and trusted sources of information related to vaccines and vaccination, so they are able to identify misinformation and thus be better informed themselves and able to correct rumors they hear from colleagues, patients and community members.

Additionally, because trust is such a critical issue for the immunization program, further interventions may be considered to address study findings under the “social processes” domain related to lack of confidence in health authorities. Such efforts might include transparent, timely communication from authorities on COVID-19 vaccination, or collaboration with trusted leaders in HCW communities who can advocate for vaccination.

For additional information on likely impact on vaccine uptake and strength of evidence, please see WHO´s “Data for action: Achieving high uptake of COVID-19 vaccines” guidance.^26^ For examples of messaging to adapt for communication strategies, see Annex D in the electronic supplement.

This study has several strengths. It was widely publicized, and available online for 50 days, casting a wide net for Caribbean HCW respondents. It was available in English and French. In Trinidad and Tobago, it was available in paper form in addition to the web-based interface. Pretest work resulted in confusing phrases being clarified before data collection. Numerous significant differences between important sub-groups lend assurance that attitudes of physicians and nurses differ, attitudes of physicians and allied health professionals differ, and attitudes of younger and older respondents differ. Free text responses were independently categorized by several teams. BeSD team members from WHO Geneva advised the categorization and interpretation of free-text responses.

The study is limited by several factors. The sample was not representative of all Caribbean HCWs and the data were analyzed as if they came from a simple random sample which carries a risk spurious significant difference. Using an online survey may have resulted in sampling bias because the participants needed access to a smartphone or computer to participate. Respondents were not required to answer any of the questions, so thorough factor analysis is not possible, and the dataset does not include responses to the hesitancy proxy question for nearly 30% of respondents. The survey was conducted in March/April 2021, and attitudes may have changed - for example, some countries with a small number of COVID-19 cases at the time of the survey later developed a second and third waves of cases, which may affect HCW risk perception. And finally, vaccine acceptance may have been exaggerated here due to social desirability bias, meaning that medical professionals may have responded in a manner likely to be viewed favorably by their peers or superiors.

## Contributors

E. Benjamin Puertas: Conceptualization; Methodology; Project administration; Supervision; Formal analysis; Writing – original draft; Writing – review & editing

Martha Velandia-Gonzalez: Conceptualization; Methodology; Project administration; Supervision; Formal Analysis; Writing – original draft; Writing – review & editing

Lauren Vulanovic: Conceptualization; Project administration; Supervision; Writing – original draft; Writing – review & editing

Lisa Bayley: Investigation; Writing – review & editing

Karen Broome: Investigation; Writing – review & editing

Claudia Ortiz: Methodology; Software; Data curation; Writing – review & editing

Nina Rise: Investigation; Writing – original draft; Writing – review & editing

Maite Vera Antelo: Investigation; Writing – review & editing

Dale A. Rhoda: Methodology; Formal analysis; Visualization; Writing – original draft; Writing – review & editing

## Disclaimer

The authors alone are responsible for the views expressed in this article and they do not necessarily represent the views, decisions, or policies of the institutions with which they are affiliated.

## Data sharing

The survey instrument appears in the electronic supplement. A restricted set of de-identified survey response data will be made available on e-mail request to the corresponding author for a period of one year after publication of this article. Demographic variables and responses to Likert-type questions will be made available from all respondents. Responses to the open-ended free-text response questions will not be shared outside the original study team.

## Declaration of interests

None.

## Appendix Supplementary materials

Image, application 1

Image, application 2

Image, application 3

Image, application 4
