# Supplementary material for: Concerns, attitudes, and intended practices of Caribbean healthcare workers concerning COVID-19 vaccination: A cross-sectional study
Source: Lancet Reg Health Am. 2022 Feb 3;9:100193. doi: 10.1016/j.lana.2022.100193 (PMC8812828; doi:10.1016/j.lana.2022.100193)
Supplement: Supplementary file 1 [file mmc1.docx]

# *Editorial disclaimer: This translation in Spanish was submitted by the authors and we reproduce it as supplied. It has not been peer reviewed. Our editorial processes have only been applied to the original abstract in English, which should serve as reference for this manuscript.*

# Resumen

# Antecedentes: El Caribe tiene una larga historia de liderazgo mundial en inmunización, y un factor que ha contribuido a este éxito ha sido el compromiso de los trabajadores de la salud en la promoción de los beneficios de las vacunas. Los trabajadores de la salud desempeñan un papel fundamental en la generación de confianza entre el público y los programas de inmunizaciones y, en general, se les cita como la fuente de información más confiable sobre vacunación. Por lo tanto. los propios trabajadores de la salud deben tener confianza en la vacunación como un bien de salud pública y ser capaces de transmitir esta confianza a quienes confían en ellos. Sin embargo, al igual que el público en general, los trabajadores de la salud desarrollan confianza a diferentes ritmos y pueden ser susceptibles a la información errónea sobre las vacunas.

# Métodos: Durante abril y mayo de 2021, la Organización Panamericana de la Salud (OPS) realizó una encuesta de métodos mixtos para evaluar las actitudes, opiniones y prácticas sobre vacunación de 1197 trabajadores de la salud en 14 países del Caribe.

# Resultados: El setenta y siete por ciento de los encuestados expresaron una clara intención de vacunarse contra la COVID-19 lo antes posible. La intención de vacunarse lo antes posible fue expresada en proporciones más bajas en enfermeras (66%) y profesionales de la salud afines (6 %) que en médicos (85%) y por encuestados más jóvenes que mayores (64% frente a 85%, respectivamente; p < 0,001 para todas estas comparaciones). A través de 32 preguntas sobre actitudes y opiniones, la reticencia a las vacunas fue expresada consistentemente por una mayor proporción de enfermeras y profesionales de la salud afines que los médicos y por los encuestados más jóvenes que los mayores.

# Interpretación: Los conocimientos de la encuesta están ayudando a la OPS a abordar las preocupaciones de los trabajadores de la salud con mensajes informativos y apoyando a los países en el desarrollo de políticas para aumentar la confianza y la cobertura de la vacuna entre los trabajadores de la salud del Caribe.

# Financiamiento: Este trabajo ha sido patrocinado por la Organización Mundial de la Salud/Organización Panamericana de la Salud, el Gobierno de Alemania y la Alianza Gavi.

# PALABRAS CLAVE: reticencia a vacunas; trabajadores de la salud; Caribe; COVID-19; encuesta; aceptación de la vacuna
