# Supplementary material for: Concerns, attitudes, and intended practices of Caribbean healthcare workers concerning COVID-19 vaccination: A cross-sectional study
Source: Lancet Reg Health Am. 2022 Feb 3;9:100193. doi: 10.1016/j.lana.2022.100193 (PMC8812828; doi:10.1016/j.lana.2022.100193)
Supplement: Supplementary file 2 [file mmc2.docx]

# *Editorial disclaimer: This translation in French was submitted by the authors and we reproduce it as supplied. It has not been peer reviewed. Our editorial processes have only been applied to the original abstract in English, which should serve as reference for this manuscript.*

**Résumé**

**Contexte:** Les Caraïbes sont depuis longtemps un leader mondial de la vaccination, et l'un des facteurs ayant contribué à ce succès a été l'engagement des agents de santé à promouvoir les avantages des vaccins. Les agents de santé jouent un rôle essentiel dans l'établissement de la confiance entre le public et le programme de vaccination et sont généralement cités comme la source d'information la plus fiable sur la vaccination. Les agents de santé eux-mêmes doivent donc avoir confiance dans la vaccination en tant que bien de santé publique et pouvoir transmettre cette confiance à ceux qui leur font confiance. Cependant, tout comme le grand public, les agents de santé développent leur confiance à des rythmes différents et peuvent être sensibles à la désinformation sur les vaccins.

**Méthodes:** En avril et mai 2021, l'Organisation panaméricaine de la santé (OPS) a mené une enquête à méthodes mixtes pour évaluer les attitudes, les opinions et le raisonnement de 1,197 agents de santé dans 14 pays des Caraïbes en matière de vaccination.

**Résultats:** Soixante-dix-sept pour cent des répondants ont exprimé leur intention claire de se faire vacciner contre la COVID-19 dès que possible. L'intention de se faire vacciner le plus tôt possible a été exprimée par des proportions plus faibles d'infirmières (66%) et de professionnels paramédicaux (62%) que de médecins (85%) et par des répondants plus jeunes que les plus âgés (64% contre 85%, respectivement ; p < 0,001 pour toutes ces comparaisons). Sur 32 questions sur les attitudes et les opinions, l'hésitation à la vaccination était systématiquement exprimée par des proportions plus élevées d'infirmières et de professionnels paramédicaux que de médecins et par des répondants plus jeunes que des répondants plus âgés.

**Interprétation:** Les enseignements de l'enquête aident l'OPS à répondre aux préoccupations des agents de santé avec des messages informatifs et à soutenir les pays dans l'élaboration de politiques pour accroître la confiance et la couverture vaccinale parmi les agents de santé des Caraïbes.

**Financement:** Ce travail a été parrainé par l'Organisation mondiale de la santé/Organisation panaméricaine de la santé, le gouvernement allemand et l'Alliance Gavi.

**MOTS-CLÉS:** hésitation à la vaccination; Les agents de santé; Caraïbes; COVID-19; sondage; acceptation du vaccin.
