# Supplementary material for: Concerns, attitudes, and intended practices of Caribbean healthcare workers concerning COVID-19 vaccination: A cross-sectional study
Source: Lancet Reg Health Am. 2022 Feb 3;9:100193. doi: 10.1016/j.lana.2022.100193 (PMC8812828; doi:10.1016/j.lana.2022.100193)
Supplement: Supplementary file 3 [file mmc3.docx]

# *Editorial disclaimer: This translation in Portuguese was submitted by the authors and we reproduce it as supplied. It has not been peer reviewed. Our editorial processes have only been applied to the original abstract in English, which should serve as reference for this manuscript.*

**Resumo**

**Antecedentes:** O Caribe tem uma longa história de ser líder global em imunização, e um fator que contribui para esse sucesso tem sido o compromisso dos profissionais de saúde em promover os benefícios das vacinas. Os profissionais de saúde desempenham um papel fundamental na construção da confiança entre o público e o programa de imunização e são geralmente citados como a fonte mais confiável de informações sobre a vacinação. Os próprios profissionais de saúde, portanto, devem estar confiantes na vacinação como um bem de saúde pública e capazes de transmitir essa confiança para aqueles que confiam neles. No entanto, assim como acontece com o público em geral, os profissionais de saúde desenvolvem confiança em diferentes proporções e podem ser suscetíveis à desinformação sobre vacinas.

**Métodos:** Durante abril e maio de 2021, a Organização Pan-Americana da Saúde (OPAS) realizou uma pesquisa de métodos mistos para avaliar atitudes, opiniões e raciocínios sobre vacinação para 1.197 profissionais de saúde em 14 países do Caribe.

**Resultados**: Setenta e sete por cento dos entrevistados expressaram clara intenção de serem vacinados para o COVID-19 o mais rápido possível.  A intenção de ser vacinado o mais rápido possível foi expressa em menores proporções por enfermeiros (66%) e por profissionais de áreas ligadas à saúde (62%) do que por médicos (85%) e por entrevistados mais jovens comparados com os mais velhos (64% vs. 85%, respectivamente; p < 0,001 para todas essas comparações). Em 32 perguntas sobre atitudes e opiniões, a hesitação vacinal foi consistentemente expressa em maiores proporções por enfermeiros e por profissionais de áreas ligadas à saúde do que por médicos e por entrevistados mais jovens do que os mais velhos.

**Interpretação:** As percepções da pesquisa estão ajudando a OPAS a lidar com as preocupações dos profissionais de saúde com mensagens informativas e apoiando os países no desenvolvimento de políticas para aumentar a confiança e a cobertura das vacinas entre os profissionais de saúde do Caribe.

**Financiamento:** *Este trabalho foi patrocinado pela* Organização Mundial da Saúde/Organização Pan-Americana da *Saúde, pelo Governo da Alemanha e pela Aliança Gavi.*

**PALAVRAS-CHAVE:** hesitação vacinal; profissionais de saúde; Caribe; COVID-19; pesquisa; aceitação de vacinas.
