# Supplementary material for: Concerns, attitudes, and intended practices of Caribbean healthcare workers concerning COVID-19 vaccination: A cross-sectional study
Source: Lancet Reg Health Am. 2022 Feb 3;9:100193. doi: 10.1016/j.lana.2022.100193 (PMC8812828; doi:10.1016/j.lana.2022.100193)
Supplement: Supplementary file 4 [file mmc4.docx]

Supplement to

Concerns, Attitudes, and Intended Practices of 
Caribbean Healthcare Workers Concerning 
COVID-19 Vaccination: A cross-sectional study

E. Benjamin Puertas^1*^, Martha Velandia-Gonzalez^2^, Lauren Vulanovic^2^, Lisa Bayley^1^, Karen Broome^1^, Claudia Ortiz^2^, Nina Rise^1^, Maite Vera Antelo^2^, Dale A. Rhoda^3^

^1^ Pan American Health Organization, Office of the Subregional Program Coordination, Caribbean, Bridgetown-Barbados.

^2^ Pan American Health Organization, Family, Health Promotion and Life Course Department, Comprehensive Family Immunization Unit, Washington, DC, USA

^3^ Biostat Global Consulting, Worthington, Ohio, USA

^*^Corresponding author: [puertasb@paho.org](mailto:puertasb@paho.org) Cel. +1 246 266-5593

Table of Contents

| Annex A | The Survey Questionnaire | Page 2 |
| --- | --- | --- |
| Annex B | Tables of respondent age, gender, and job category | Page 5 |
| Annex C | Summary of Responses Including Colored Bars and Chi-Square and Logistic Regression P-Values, by Question | Page 6 |
| Annex D | Messaging for Caribbean Healthcare Workers Based on Survey Results | Page 39 |
| Annex E | Exploratory Factor Analysis | Page 45 |

## Annex A. Questionnaire

|  | 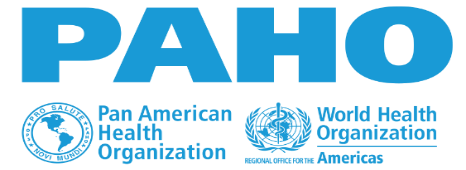   \|  \| \| --- \| |  |  |  | |  | | |  |
| --- | --- | --- | --- | --- | --- | --- | --- | --- | --- | --- |
|  | **CONCERNS, ATTITUDES, AND INTENDED PRACTICES OF HEALTHCARE WORKERS TO COVID-19 VACCINE IN THE CARIBBEAN** | | | | | |  |  |  |
|  |  | | | | | |  |  |  |
|  | Thank you very much for participating in this survey for healthcare workers. The questionnaire has a duration of no more than eight (8) minutes.  No. _______ | | | | | |  |  |  |
|  |  |  |  |  |  | |  |  |  |
| 1 | **Country where you work: ______________** |  |  |  |  | |  |  |  |
| 2 | \| **Sex: Male Female Other** \| \| --- \| | 3. **Age: _____** | | | | |  |  |  |
| 4 | **Job title/Post: ___________________** | 5. **HCW Category:** | |  |  | |  |  |  |
| Please choose the box with the response that best fits your personal concerns, attitudes and intended practices: | | | | | | |  |  |  |
|  |  | **Strongly Agree** | **Agree** | **Disagree** | **Strongly Disagree** | | |  |  |
|  | **Attitudes to vaccines** |  |  |  |  | | |  |  |
| 6 | Vaccines are important for my health |  |  |  |  | | |  |  |
| 7 | Getting vaccines is a good way to protect myself from disease |  |  |  |  | | |  |  |
| 8 | Overall, vaccines are safe |  |  |  |  | | |  |  |
| 9 | Overall, vaccines are effective |  |  |  |  | | |  |  |
| 10 | Getting vaccinated is important for the health of others in my community |  |  |  |  | | |  |  |
| 11 | The information I receive about vaccines from public health authorities/ my healthcare provider is reliable and trustworthy |  |  |  |  | | |  |  |
| 12 | Generally, I do what my doctor or health care provider recommends about vaccines for myself and my family |  |  |  |  | | |  |  |
|  |  |  |  |  |  | |  |  |  |
|  | **Vaccine readiness** | **Strongly Agree** | **Agree** | **Disagree** | **Strongly Disagree** | | |  |  |
| 13 | New vaccines carry more risk than older vaccines |  |  |  |  | | |  |  |
| 14 | I would recommend a COVID-19 vaccine to friends and family |  |  |  |  | | |  |  |
| 15 | I am concerned about serious adverse effects of vaccines |  |  |  |  | | |  |  |
|  |  |  |  |  |  | |  |  |  |
|  | **Attitudes towards COVID-19 vaccine** | **Strongly Agree** | **Agree** | **Disagree** | **Strongly Disagree** | | |  |  |
| 16 | A coronavirus (COVID−19) vaccine will protect me from severe COVID disease |  |  |  |  | | |  |  |
| 17 | I am confident in the scientific approval process for a new coronavirus (COVID−19) vaccine |  |  |  |  | | |  |  |
| 18 | I would be willing to participate in a vaccine trial for a coronavirus (COVID−19) vaccine |  |  |  |  | | |  |  |
|  | *If a new coronavirus (COVID−19) vaccine becomes publicly available:* |  | | | | |  |  |  |
| 19 | I intend to get it as soon as possible |  |  |  |  | | |  |  |
| 20 | I intend to wait to see how it affects others before I get it |  |  |  |  | | |  |  |
| 21 | I do not intend on getting it soon, but might sometime in the future |  |  |  |  | | |  |  |
| 22 | I do not intend to ever get the vaccine |  |  |  |  | | |  |  |
|  |  |  |  |  |  | | |  |  |
|  | **Please indicate how you feel about the statements below** | **Strongly Agree** | **Agree** | **Disagree** | **Strongly Disagree** | | |  |  |
| 23 | I am confident there will be other effective treatments soon |  |  |  |  | | |  |  |
| 24 | I do not yet know enough about the vaccine to make a decision |  |  |  |  | | |  |  |
| 25 | I want to gain natural immunity to the virus that causes COVID−19 |  |  |  |  | | |  |  |
| 26 | Development of the vaccine may be rushed/the vaccine may not be thoroughly tested prior to approval |  |  |  |  | | |  |  |
| 27 | I believe vaccines may give you the disease they are designed to protect against |  |  |  |  | | |  |  |
| 28 | Other reasons for delaying or refusing COVID-19 vaccine: | | | | | |  |  |  |
|  |  |  |  |  |  | | |  |  |
|  | **Attitudes towards COVID-19 vaccine** |  |  |  |  | | |  |  |
|  | **The following factors contributed to my opinion on a COVID−19 vaccine:** | **Strongly Agree** | **Agree** | **Disagree** | **Strongly Disagree** | | |  |  |
| 29 | The pace at which the vaccine was researched and developed |  |  |  |  | | |  |  |
| 30 | The unfolding & frequently evolving science of SARS−CoV−2 |  |  |  |  | | |  |  |
| 31 | Actions and opinions of my friends and family regarding the vaccine |  |  |  |  | | |  |  |
| 32 | The relationship between coverage rates and community transmission |  |  |  |  | | |  |  |
| 33 | My own research on COVID−19 vaccines |  |  |  |  | | |  |  |
| 34 | The country in which a vaccine is manufactured |  |  |  |  | | |  |  |
| 35 | The potential cost of a COVID−19 vaccine |  |  |  |  | | |  |  |
| 36 | Information I’ve seen on social media. |  |  |  |  | | |  |  |
| 37 | Other factors: | | | | | |  |  |  |
|  |  |  |  |  |  | | |  |  |
|  | **Attitudes towards influenza vaccine** | **Strongly Agree** | **Agree** | **Disagree** | **Strongly Disagree** | | |  |  |
| 38 | I would take the flu vaccine if offered |  |  |  |  | | |  |  |
| 39 | If you disagree, what are the reasons why? __________________________________________ | | | | | |  |  |  |
|  |  | **Strongly Agree** | **Agree** | **Disagree** | **Strongly Disagree** | | |  |  |
| 40 | I would recommend the flu vaccine to friends and family |  |  |  |  | | |  |  |
| 41 | If you disagree, what are the reasons why? _____________________________________________ | | | | | |  |  |  |

Thanks again for your participation!

Please, feel free to share this survey with other healthcare workers who may be interested in participating.

## Annex B. Tables of respondent age, gender, and job category

Table B-1. Survey respondents by gender and job category; these appear graphically in Figure 1.

|  | Physicians | Nurses | Public Health | Allied Pros | Other |
| --- | --- | --- | --- | --- | --- |
| Female | 343 | 311 | 82 | 116 | 50 |
| Male | 174 | 17 | 34 | 41 | 22 |
| Other | 3 | 1 | 0 | 1 | 0 |
| Missing | 1 | 1 | 0 | 0 | 0 |

Table B-2. Survey respondents by age quartile and job category; these appear graphically in Figure 1.

|  | Physicians | Nurses | Public Health | Allied Pros | Other |
| --- | --- | --- | --- | --- | --- |
| 21-32 years | 153 | 81 | 16 | 45 | 14 |
| 33-40 years | 136 | 66 | 29 | 40 | 19 |
| 41-50 years | 106 | 87 | 43 | 43 | 17 |
| 51-87 years | 117 | 94 | 27 | 27 | 18 |
| Missing Age | 9 | 2 | 1 | 3 | 4 |

## Annex C. Summary of Responses Including Colored Bars and Chi-Square and Logistic Regression P-Values, by Question

Each table in this annex summarizes responses to a single survey question. The rows represent subgroups of respondents. The first four columns summarize the proportion who answered Strongly agree, Agree, Disagree, and Strongly disagree. The next two columns consolidate the responses into two categories: Strongly agree and Agree versus Disagree and Strongly disagree. The next column indicates the number of persons in each subgroup who responded to the question. The next column lists chi-square *p*-values that test the hypothesis that the percentage who Strongly agree or Agree is the same:

1. Between nurses and physicians
2. Across all categories of healthcare workers
3. Among categories of physicians
4. Among categories of nurses
5. Among care categories
6. Between men and women
7. Among age quartiles.

*P*-values smaller than 0.05 are listed in a bold font and indicate a statistically significant difference.

The final four columns show results from multivariable logistic regression, where the outcome is 1 if the respondent selected Strongly agree or Agree and is 0 if they selected Disagree or Strongly disagree. The regression uses three categorical predictors: healthcare worker category (physician is the reference group); sex (male is the reference); and age quartile (youngest is the reference group). Each table lists odds ratios, *p*-values, and 95% confidence intervals for the odds ratios. *P*-values smaller than 0.05 are listed in a bold font and indicate a statistically significant result when simultaneously adjusting for differences in job category, sex, and age.

**
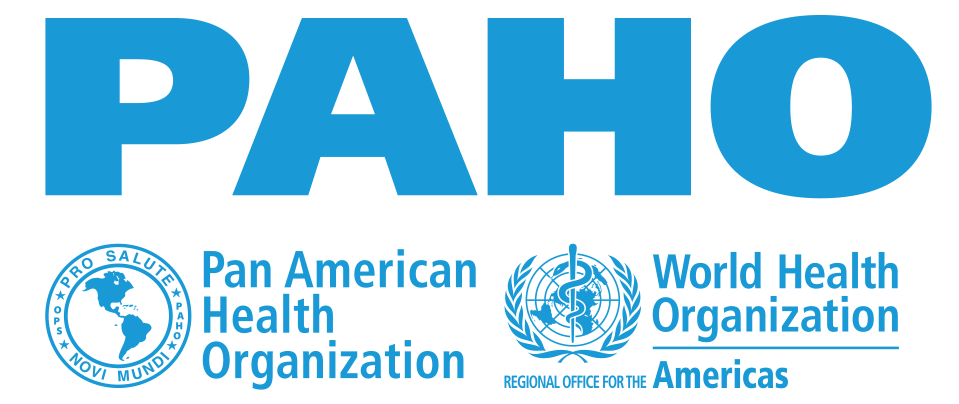
**

## Annex D. Messaging for Caribbean Healthcare Workers Based on Survey Results

***Concerns, Attitudes, and Intended Practices of HCWs to COVID-19 Vaccination in the Caribbean***

**Messaging for Caribbean Healthcare Workers Based on Survey Results**

| **Concern** | **Objective** | **Topline messages** | **Supporting messages** |
| --- | --- | --- | --- |
| The vaccine doesn’t work, I’ll have to continue with protective measures anyway. | - Reinforce efficacy of vaccines in preventing severe illness and death. - Remind public health measures aren’t for forever. | - All vaccines against COVID-19 that have been listed by WHO are extremely safe and effective at preventing death and severe illness. - WHO recommends that people who are fully vaccinated should continue to follow public health measures like masking and physical distancing until more people are protected against COVID-19. - Vaccination for everyone who is eligible is key to ending the pandemic. | - All vaccines go through clinical trial phases with tens of thousands of people of different ages and ethnicities before they are approved for use in the population. These trials aim to ensure the safety and ability of the vaccine to protect against the disease. - Only those vaccines that are safe and have proven to be effective at preventing the disease are approved to be used in the population. - Although data show us that vaccines work well in preventing people from getting sick, researchers are still learning about how well the vaccines stop the spread of COVID-19 between people. This is why WHO still recommends public health measures that have been used since the start of the pandemic to stop the spread of the virus. - Real-world data are showing us that the vaccines are extremely effective and safe. |
| The vaccines were developed too quickly and we don’t know enough about them yet. | - Reinforce safety of vaccines. - Remind that corners were not cut and data are reliable. | - Vaccine safety is always a top priority, and this is no different for COVID-19 vaccines. - Unprecedented collaboration between researchers and partners at the global level allowed COVID-19 vaccines to be developed quickly. - Red tape was cut, but not corners. - The technology used to develop these vaccines has been used for decades for other treatments that we use routinely. | - All vaccines – including those against COVID-19 – go through clinical trial phases with tens of thousands of people of different ages and ethnicities before they are approved for use in the population. These trials aim to ensure the safety and ability of the vaccine to protect against the disease. - The COVID-19 vaccines were not created overnight, but rather were developed following decades of research on other coronaviruses like SARS and MERS, and using technology that has long been used for other medical treatments (like mRNA platforms being used for cancer treatment). - Because of the seriousness of the pandemic, developing vaccines against COVID-19 has been a global priority. - Real-world data are showing us that the vaccines are extremely effective and safe. |
| I’m scared of side effects from the vaccines, especially long term. | - Reinforce safety of vaccines. - Remind that corners were not cut and data are reliable. | - Minor side effects are normal with any vaccine and go away after a few days. - Medical researchers have determined that the benefits of the vaccine far outweigh the minor possibility of potentially associated serious risks. | - Real-world data and follow-up of people who participated in the clinical trials for the vaccines show that the vaccines are extremely effective and safe. - There is a small risk of a serious side effect for any vaccine, just like there are risks of side effects for any medication. - After you are vaccinated, the components of the vaccine are broken down by your body quickly; the vaccine does not linger in your body and cannot cause long-term damage. - The vaccines do not enter your cells’ nuclei and cannot alter your DNA, causing long-term damage.^[[1]](#footnote-1)^ |
| I don’t want the vaccine that’s available to me/ I don’t trust the vaccine manufacturer. | - Reinforce efficacy of vaccines in preventing severe illness and death. | - The best vaccine is the one that is available to you first. - Data have shown us that **all** approved vaccines are extremely safe and effective at preventing serious disease and death. - Waiting for your “favorite” or preferred vaccine puts you at risk for longer. | - The sooner you are vaccinated with any of the approved vaccines, the sooner you can be protected from a serious case of COVID-19 and death. - All of the approved vaccines can help us fight the pandemic. - All vaccine manufacturers must follow the same processes for getting authorization by WHO; there are no exceptions. This includes presenting detailed data on their safety and efficacy. Only vaccines that have proved these will be approved. |
| I don’t trust my government’s/ WHO’s handling of the pandemic and the vaccine rollout.^[[2]](#footnote-2)^ | - Reinforce global collaboration efforts for the development of the vaccines. - Show personal reasons why trusted individuals have chosen to be vaccinated. | - Data have shown us that **all** approved vaccines are extremely safe and effective at preventing serious disease and death. - Safety monitoring continues after vaccines are introduced in the population. - The vaccines are a result of global collaboration to fight the pandemic. | - For main and supporting messages, suggest using a trusted leader (according to the target audience) to deliver them. - For supporting messages, suggest adding personal reasons why this leader chose to get vaccinated. |
| I don’t need a vaccine because my religion will protect me. | - Remind people of the moral obligation to get vaccinated to protect others. | - Getting vaccinated is the most effective way to protect you from COVID-19. - Getting vaccinated helps your community by keeping health services functioning. - We have a moral duty to get vaccinated. | - Health services have been overwhelmed because of the pandemic. When fewer people are hospitalized due to COVID-19, health services can focus on providing other services. - The socioeconomic effects of the pandemic have also hurt people, including by worsening their health. - Getting vaccinated is doing our part to protect our communities, help our health systems, and support people’s individual economies. - Note: Include specific messages using religious denomination to explain the need for COVID-19 vaccines. |
| COVID-19 is not a problem in my country. We don’t need a vaccine. | - Increase risk perception for COVID-19 at the individual and health system level. | - In a globalized world, it is very easy for disease to spread across country borders. - COVID-19 will not be defeated until everyone is safe. - Our public health response must not leave anyone or any country behind. | - PAHO encourages people to get vaccinated against COVID-19 with whichever vaccine is offered to them by their national health authorities when they are eligible. |
| I have allergies. | - Clarify that people with allergies can still be vaccinated. | - People with allergies can take the COVID-19 vaccine unless they have had a severe allergic reaction (anaphylaxis) to any component of the COVID-19 vaccine. - Anyone with severe allergic reactions to foods, oral medications, latex, pets, insects, and environmental triggers may still get vaccinated against COVID-19. | - People with a severe allergic reaction (anaphylaxis) to any vaccine or injectable (intramuscular or intravenous) medication should consult with their health provider to assess risk prior to receiving the COVID-19 vaccine. - People with severe allergies require a 30-minute observation period after vaccination, while all others must be observed for 15 minutes. Vaccine clinics have safety protocols in place to respond to any adverse reactions. - Only a very small percentage of the population is severely allergic to the components of the COVID-19 vaccines. |
| I’m breastfeeding. | - Clarify that breastfeeding persons can still be vaccinated. | - There is no contraindication for breastfeeding persons to be vaccinated against COVID-19. - The vaccines against COVID-19 are safe for both the breastfeeding person and the child. | - Persons who are breastfeeding can still get vaccinated against COVID-19 when it is their turn. - Discontinuing breastfeeding is not recommended, as this offers substantial health benefits to lactating women and their breastfed children. |
| I’m pregnant. | - Clarify guidance on pregnancy and vaccination.^[[3]](#footnote-3)^ | - Vaccination against COVID-19 is recommended for pregnant women. - There is no evidence that suggests vaccination would cause harm to the mother or unborn baby during pregnancy. | - Pregnant women are at risk of contracting COVID-19, but because their immune systems change throughout pregnancy, pregnant women are more vulnerable to respiratory infections such as COVID-19. - If pregnant women do become ill, they tend to develop more severe symptoms whose treatment may require longer hospitalization in intensive care units, greater need for ventilatory support, and a higher chance of dying when compared with non-pregnant women of the same age and ethnicity. - Note: This messaging may need to be adjusted based on the country’s guidelines. |
| I have chronic disease or other health issues. | - Clarify that people with health issues can still be vaccinated. | - [Vaccines have been found to be safe and effective in people with various underlying medical conditions](https://www.youtube.com/watch?v=I8Remuje4bs&list=PL9S6xGsoqIBXHSDMCp8CjOmhULeQnJ_7J&index=9) that are associated with increased risk of severe disease. These include high blood pressure; diabetes; asthma; pulmonary, liver, or kidney disease; and chronic infections that are stable and controlled. - People with chronic conditions are at higher risk for complications from COVID-19 and should get vaccinated as soon as they can. | - COVID-19 vaccines have been tested in large, randomized controlled trials that include people of a broad age range, both sexes, different ethnicities, and those with known medical conditions. The vaccines have shown a high level of efficacy across all populations. - Those who should consult with a doctor before vaccination include people with a compromised immune system, older people with severe frailty, people with a history of severe allergic reaction to vaccines, people living with HIV who have weakened immune systems,^[[4]](#footnote-4)^ and those who are pregnant or breastfeeding. |
| I’ve already had COVID-19, I don’t need a vaccine. | - Clarify that people who have had COVID-19 should still be vaccinated. | - [People who have already been infected with SARS-CoV-2 should still get vaccinated](https://www.youtube.com/watch?v=L2UUBQSOVDc&list=PL9S6xGsoqIBXHSDMCp8CjOmhULeQnJ_7J&index=11) unless told otherwise by their health care provider. | - It is still not known how long the immunity from the disease lasts. - Some people get infected with SARS-CoV-2 a second time, which makes getting vaccinated even more important. - Even if you had a previous infection, the vaccine acts as a booster that strengthens the immune response. |

## Annex E. Exploratory factor analysis

**Background**

One of the manuscript reviewers, Prof. Chris McManus from University College London, suggested that rather than use a single question (Q19) as a proxy for COVID-19 vaccine hesitancy, it would be interesting “to know more about the factorial structure and dimensionality of the questionnaire. It might be helpful in the Supplementary information to have the results of a factor analysis, probably using a Varimax Rotation and extracting factors using a scree-slope or similar criterion, so that one can see the loadings of questions on the separate factors.” This annex explores those issues. These analyses were conducted post-hoc. They were not part of the original analysis plan, but the results are interesting, and the ideas could possibly influence design choices in upcoming surveys, so we include them here and express our gratitude to Prof. McManus.

A straightforward factor analysis is rendered impossible by the fact that the survey instrument did not require respondents to answer any of the questions. Setting aside the final two question about influenza vaccine, only 97 of the 1,097 respondents answered all of the 30 Likert type questions.

**Methods**

We follow the approach documented by the UCLA Statistical Consulting Group to use maximum likelihood estimation with the expectation maximation algorithm to estimate the covariance matrix among non-missing responses and then conduct factor analysis on that matrix.^1–4^ A scree plot is employed to show the eigenvalues and help decide how many factors to retain for varimax rotation. Five factors were retained and rotated and the loadings are reported and described.

**Results**

Figure E1 shows that five factors yield eigenvalues greater than one, so factor analysis was repeated extracting only five factors which were then summarized after varimax rotation. Table E1 shows the factor loadings with blank entries where the absolute value is smaller than 0.5. Table E2 shows all of the loadings. Table E3 reminds the reader briefly of the text of the relevant questions and lists the number of completed responses to each question.

**Figure E1. Screeplot of factor analysis eigenvalues of Likert type questions Q6-Q36**
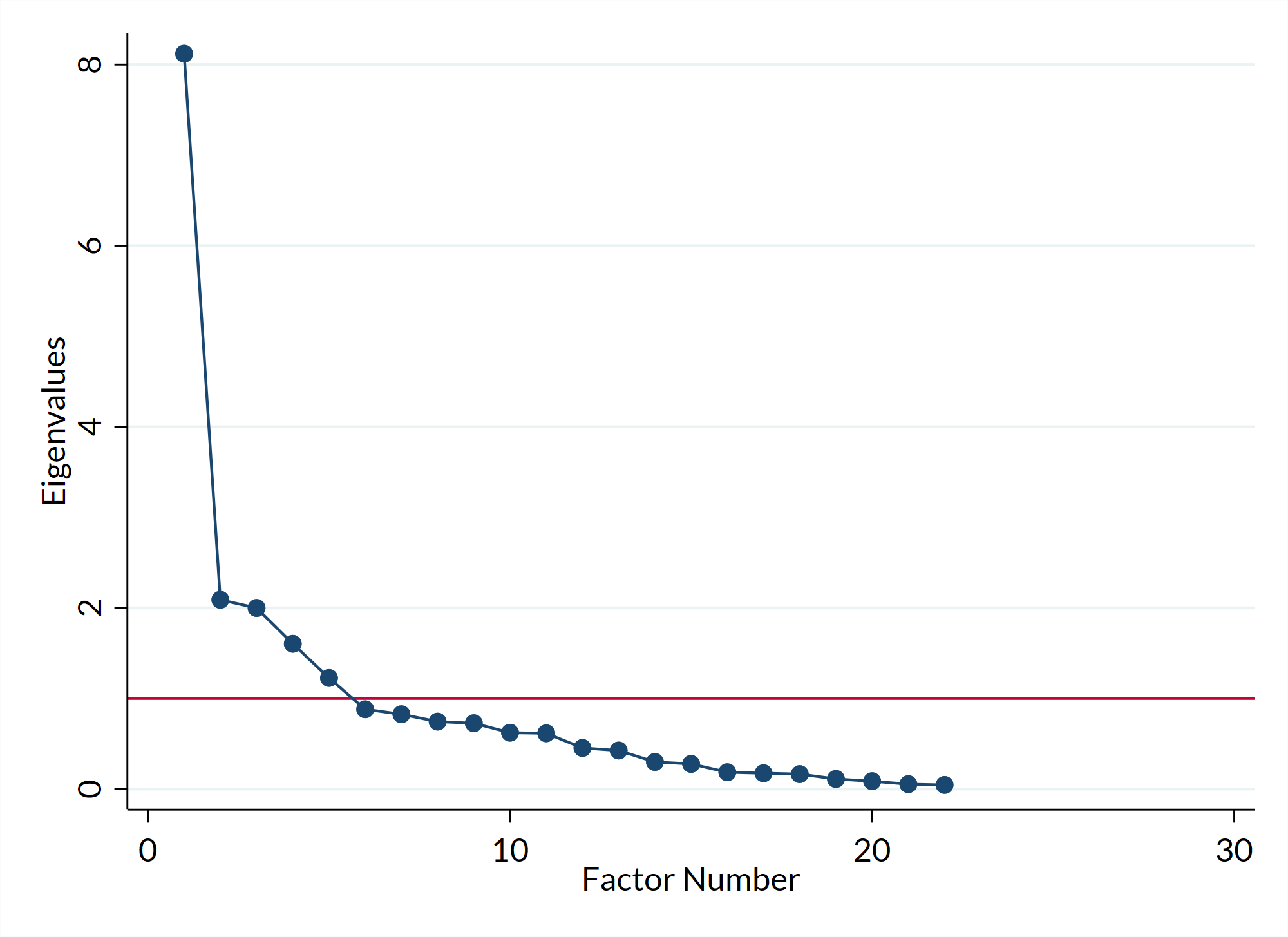


Note: The red line at y=1 represents a common threshold for identifying the number of notable factors.

**Table E1. Factor loadings with absolute value ≥ 0.5**

Factor analysis/correlation Number of obs = 97

Method: maximum likelihood Retained factors = 5

Rotation: orthogonal varimax (Kaiser off) Number of params = 140

Schwarz's BIC = 766.691

Log likelihood = -63.11568 (Akaike's) AIC = 406.231

--------------------------------------------------------------------------

Factor | Variance Difference Proportion Cumulative

-------------+------------------------------------------------------------

Factor1 | 6.00922 1.94587 0.3829 0.3829

Factor2 | 4.06335 1.79115 0.2589 0.6418

Factor3 | 2.27220 0.24053 0.1448 0.7865

Factor4 | 2.03167 0.71316 0.1294 0.9160

Factor5 | 1.31851 . 0.0840 1.0000

--------------------------------------------------------------------------

LR test: independent vs. saturated: chi2(435) = 1445.06 Prob>chi2 = 0.0000

LR test: 5 factors vs. saturated: chi2(295) = 107.80 Prob>chi2 = 1.0000

Rotated factor loadings (pattern matrix) and unique variances

-------------------------------------------------------------------------------

Variable | Factor1 Factor2 Factor3 Factor4 Factor5 | Uniqueness

-------------+--------------------------------------------------+--------------

q6 | 0.8526 | 0.2063

q7 | 0.8159 | 0.2330

q8 | 0.7033 | 0.2208

q9 | 0.6551 | 0.2982

q10 | 0.7463 | 0.2870

q11 | | 0.4127

q12 | | 0.4979

q13 | 0.5158 | 0.6287

q14 | -0.6676 | 0.2972

q15 | | 0.6882

q16 | -0.5892 | 0.3646

q17 | -0.6817 | 0.3050

q18 | -0.6038 | 0.5560

q19 | -0.7266 | 0.2871

q20 | 0.6860 | 0.4441

q21 | 0.6866 | 0.4050

q22 | | 0.5665

q23 | | 0.9457

q24 | 0.6865 | 0.3594

q25 | | 0.5077

q26 | 0.6918 | 0.3577

q27 | | 0.5707

q29 | | 0.4741

q30 | 0.6792 | 0.5031

q31 | 0.5457 | 0.6021

q32 | 0.5161 | 0.6805

q33 | | 0.8232

q34 | 0.5542 | 0.6303

q35 | 0.6075 | 0.6115

q36 | 0.5954 | 0.5406

-------------------------------------------------------------------------------

(blanks represent abs(loading)<.5)

**Factor 1.** The first factor embodies the idea of COVID-19 vaccine hesitancy. Its loadings have absolute value > 0.5 for the following questions, with positive loadings for statements that express hesitancy and negative for those that express readiness to use a COVID-19 vaccine.

Q13 "Readiness: New vaccines carry more risk than older vaccines"

Q14 "Readiness: I would recommend a COVID-19 vaccine to friends and family"

Q16 "COVID-19: A COVID−19 vaccine will protect me from severe COVID disease"

Q17 "COVID-19: I am confident in the COVID-19 vaccine scientific approval process"

Q18 "COVID-19: I would be willing to participate in a COVID-19 vaccine trial "

Q19 "COVID-19 vaccine: I intend to get it as soon as possible"

Q20 "COVID-19 vaccine: I intend to wait to see how it affects others before I get it"

Q21 "COVID-19 vaccine: I do not intend to get it soon, but might in the future"

Q24 "Reasons: I do not yet know enough about the vaccine to make a decision"

Q26 "Reasons: Development may be rushed/vaccine may not be thoroughly tested"

**Factor 2.** The second factor loads heavily on the following questions, embodying the idea that vaccines in general are good for personal health:

Q6 "Attitudes: Vaccines are important for my health"

Q7 "Attitudes: Vaccines are a good way to protect myself from disease"

Q10 "Attitudes: Vaccines are important for the health of others"

**Factor 3.** The third factor loads heavily on four statements that address apparently concerns that could contribute to hesitancy, but are possibly less personal than some of the others.

Q31 "Opinion shapers: Actions and opinions of friends and family"

Q34 "Opinion shapers: The country in which a vaccine is manufactured"

Q35 "Opinion shapers: The potential cost of a COVID−19 vaccine"

Q36 "Opinion shapers: Information I´ve seen on social media"

**Factor 4.** The fourth factor loads heavily on these less explicitly health-related statements about vaccination in general:

Q8 "Attitudes: Vaccines are safe"

Q9 "Attitudes: Vaccines are effective"

**Factor 5.** The fifth factor loads on two statements that reflect an interest in the science of COVID-19

Q30 "Opinion shapers: The unfolding & frequently evolving science of SARS−CoV−2"

Q32 "Opinion shapers: Relationship between coverage rates and community transmission"

Note: All loadings with absolute value ≥ 0.5 for factors 2-5 are positive.

**Table E2. Full set of rotated factor loadings**

Factor analysis/correlation Number of obs = 97

Method: maximum likelihood Retained factors = 5

Rotation: orthogonal varimax (Kaiser off) Number of params = 140

Schwarz's BIC = 766.691

Log likelihood = -63.11568 (Akaike's) AIC = 406.231

--------------------------------------------------------------------------

Factor | Variance Difference Proportion Cumulative

-------------+------------------------------------------------------------

Factor1 | 6.00922 1.94587 0.3829 0.3829

Factor2 | 4.06335 1.79115 0.2589 0.6418

Factor3 | 2.27220 0.24053 0.1448 0.7865

Factor4 | 2.03167 0.71316 0.1294 0.9160

Factor5 | 1.31851 . 0.0840 1.0000

--------------------------------------------------------------------------

LR test: independent vs. saturated: chi2(435) = 1445.06 Prob>chi2 = 0.0000

LR test: 5 factors vs. saturated: chi2(295) = 107.80 Prob>chi2 = 1.0000

Rotated factor loadings (pattern matrix) and unique variances

-------------------------------------------------------------------------------

Variable | Factor1 Factor2 Factor3 Factor4 Factor5 | Uniqueness

-------------+--------------------------------------------------+--------------

q6 | -0.2256 0.8526 -0.0457 0.1172 0.0115 | 0.2063

q7 | -0.2158 0.8159 -0.0660 0.2232 -0.0221 | 0.2330

q8 | -0.2772 0.4486 -0.0793 0.7033 0.0080 | 0.2208

q9 | -0.2151 0.4468 -0.1631 0.6551 -0.0095 | 0.2982

q10 | -0.2879 0.7463 -0.0654 0.2575 0.0511 | 0.2870

q11 | -0.3788 0.4466 0.0178 0.4929 0.0334 | 0.4127

q12 | -0.3479 0.3889 0.0794 0.4717 0.0312 | 0.4979

q13 | 0.5158 -0.2061 0.1451 -0.1293 0.1581 | 0.6287

q14 | -0.6676 0.4096 0.0376 0.2876 -0.0723 | 0.2972

q15 | 0.4676 -0.1576 0.1880 -0.1366 0.1197 | 0.6882

q16 | -0.5892 0.4577 0.0061 0.2747 -0.0563 | 0.3646

q17 | -0.6817 0.3306 0.0500 0.3367 -0.0712 | 0.3050

q18 | -0.6038 0.1691 0.1188 0.1874 -0.0403 | 0.5560

q19 | -0.7266 0.3786 0.0513 0.1963 -0.0188 | 0.2871

q20 | 0.6860 -0.1324 0.2591 -0.0171 0.0209 | 0.4441

q21 | 0.6866 -0.1238 0.3223 -0.0400 -0.0524 | 0.4050

q22 | 0.4155 -0.4163 0.2522 -0.1354 -0.0747 | 0.5665

q23 | 0.0806 0.0081 0.2041 -0.0695 0.0345 | 0.9457

q24 | 0.6865 -0.2120 0.3117 -0.1646 -0.0147 | 0.3594

q25 | 0.4516 -0.3605 0.3801 -0.1178 -0.0021 | 0.5077

q26 | 0.6918 -0.2535 0.2304 -0.1212 0.1780 | 0.3577

q27 | 0.3816 -0.3636 0.3442 -0.1382 0.1183 | 0.5707

q29 | 0.4795 -0.1053 0.2531 -0.0398 0.4682 | 0.4741

q30 | 0.1378 0.0538 0.1147 0.0217 0.6792 | 0.5031

q31 | 0.1917 -0.0839 0.5457 -0.0993 0.2155 | 0.6021

q32 | -0.0338 0.0492 0.2224 -0.0110 0.5161 | 0.6805

q33 | -0.1432 0.0784 -0.0110 0.0844 0.3780 | 0.8232

q34 | 0.1030 -0.0124 0.5542 0.0365 0.2246 | 0.6303

q35 | 0.0261 0.0162 0.6075 -0.0819 0.1089 | 0.6115

q36 | 0.2596 -0.1690 0.5954 -0.0701 0.0632 | 0.5406

-------------------------------------------------------------------------------

**Table E2, continued.**

Factor rotation matrix

-----------------------------------------------------------

| Factor1 Factor2 Factor3 Factor4 Factor5

-------------+---------------------------------------------

Factor1 | -0.6596 0.6202 -0.1713 0.3844 -0.0572

Factor2 | 0.5258 0.5422 0.4770 0.2909 0.3426

Factor3 | -0.5194 -0.3302 0.7328 0.0112 0.2901

Factor4 | 0.1242 -0.4425 -0.0720 0.8751 -0.1330

Factor5 | 0.0575 0.1286 0.4484 -0.0402 -0.8818

-----------------------------------------------------------

**Table E3. Abbreviated Likert Question Text and Number of Response**

| Abbreviated Question Text | Number of Completed Responses |
| --- | --- |
| Q6 Attitudes: Vaccines are important for my health | 1,159 |
| Q7 Attitudes: Vaccines are a good way to protect myself from disease | 1,143 |
| Q8 Attitudes: Vaccines are safe | 1,075 |
| Q9 Attitudes: Vaccines are effective | 1,081 |
| Q10 Attitudes: Vaccines are important for the health of others | 1,143 |
| Q11 Attitudes: Vaccine information is reliable and trustworthy | 1,031 |
| Q12 Attitudes: I do what my care provider recommends about vaccines | 1,144 |
| Q13 Readiness: New vaccines carry more risk than older vaccines | 822 |
| Q14 Readiness: I would recommend a COVID-19 vaccine to friends and family | 1,008 |
| Q15 Readiness: I am concerned about serious adverse effects of vaccines | 1,129 |
| Q16 COVID-19: A COVID−19 vaccine will protect me from severe COVID disease | 909 |
| Q17 COVID-19: I am confident in the COVID-19 vaccine scientific approval process | 892 |
| Q18 COVID-19: I would be willing to participate in a COVID-19 vaccine trial | 815 |
| Q19 COVID-19 vaccine: I intend to get it as soon as possible | 848 |
| Q20 COVID-19 vaccine: I intend to wait to see how it affects others before I get it | 1,008 |
| Q21 COVID-19 vaccine: I do not intend to get it soon, but might in the future | 996 |
| Q22 COVID-19 vaccine: I do not intend to ever get the vaccine | 965 |
| Q23 Reasons: I am confident there will be other effective treatments soon | 682 |
| Q24 Reasons: I do not yet know enough about the vaccine to make a decision | 1,039 |
| Q25 Reasons: I want to gain natural immunity to the virus that causes COVID−19 | 964 |
| Q26 Reasons: Development may be rushed/vaccine may not be thoroughly tested | 916 |
| Q27 Reasons: I believe vaccines may give you the disease | 955 |
| Q29 Opinion shapers: The pace at which the vaccine was researched and developed | 972 |
| Q30 Opinion shapers: The unfolding & frequently evolving science of SARS−CoV−2 | 932 |
| Q31 Opinion shapers: Actions and opinions of friends and family | 958 |
| Q32 Opinion shapers: Relationship between coverage rates & community transmission | 723 |
| Q33 Opinion shapers: My own research on COVID−19 vaccines | 960 |
| Q34 Opinion shapers: The country in which a vaccine is manufactured | 904 |
| Q35 Opinion shapers: The potential cost of a COVID−19 vaccine | 854 |
| Q36 Opinion shapers: Information I´ve seen on social media. | 950 |
| Q38 Influenza: I would take the flu vaccine if offered | 958 |
| Q40 Influenza: I would recommend the flu vaccine to friends and family | 932 |

**Discussion**

If all respondents had answered all questions, it would seem prudent to use Factor 1 to characterize vaccine hesitancy instead of Q19 alone. The factor loads on two issues of readiness, all the questions about timing of willingness to be vaccinated, and two reasons to delay vaccination. This factor would be more robust than a single proxy question. Because of the large number of missing responses documented in Table E3, without a large amount of imputation, it would only be possible to calculate the factor scores for fewer than 10% of respondents. In this manuscript we choose to keep the focus on summarizing the data from questions that respondents did choose to answer but note for those who may be planning future similar surveys that it is worthwhile to consider requiring responses to all questions in order to facilitate insightful and robust factor analysis.

**References**

^1^ UCLA: Statistical Consulting Group. How can I do factor analysis with missing data in Stata? https://stats.idre.ucla.edu/stata/faq/how-can-i-do-factor-analysis-with-missing-data-in-stata/.

^2^ Graham JW. Missing Data Analysis: Making It Work in the Real World. *Annu Rev Psychol* 2009; **60**: 549–76.

^3^ Truxillo C. Maximum Likelihood Parameter Estimation with Incomplete Data. In: Proceedings of the Thirtieth Annual SAS Users Group International (SUGI) Conference. Philadelphia, Pennsylvania, USA, 2005: 19.

^4^ Weaver B, Maxwell H. Exploratory factor analysis and reliability analysis with missing data: A simple method for SPSS users. *TQMP* 2014; **10**: 143–52.

1. This bullet and the one before it are in response to qualitative answers expressing concerns about the vaccines causing long-term effects, potentially through entering people’s DNA. [↑](#footnote-ref-1)
2. Suggest that these messages be shared via trusted spokespersons (local leaders, community health workers, religious leaders, local media, other healthcare workers, etc.). [↑](#footnote-ref-2)
3. See more information at: <https://www.who.int/news-room/events/detail/2021/05/05/default-calendar/update-on-covid-19-vaccination-in-pregnant-women-and-children> [↑](#footnote-ref-3)
4. People living with HIV (PLHIV) have been included in the clinical trials for four of the five vaccines with EUL approval (the exception is Sinopharm). All five are recommended for PLHIV. Vaccine efficacy for this population is comparable to that found among HIV-negative persons. Safety data are scarce and not specific to PLHIV. Nonetheless, the benefits of vaccination far outweigh the risks. Unless PLHIV have a weakened immune system (i.e., poorly controlled CD4 count), there are no recommendations to consult a physician before receiving a COVID-19 vaccine. [↑](#footnote-ref-4)
